# The Relationship between PID-5 Personality Traits and Mental States. A Study on a Group of Young Adults at Risk of Psychotic Onset

**DOI:** 10.3390/medicina57010033

**Published:** 2021-01-01

**Authors:** Maria Meliante, Chiara Rossi, Lara Malvini, Clara Niccoli, Osmano Oasi, Simona Barbera, Mauro Percudani

**Affiliations:** 1 Department of Mental Health and Addiction Services, Niguarda Hospital, Milan (I), Piazza Ospedale Maggiore, 3, 20162 Milano, Italy; maria.meliante@ospedaleniguarda.it (M.M.); lara.malvini@ospedaleniguarda.it (L.M.); simona.barbera@ospedaleniguarda.it (S.B.); 2 Department of Psychology, Catholic University of Milan (I), Largo Agostino Gemelli, 1, 20123 Milano, Italy; chiara.rossi1@unicatt.it (C.R.); clara.niccoli@gmail.com (C.N.); osmano.oasi@unicatt.it (O.O.)

**Keywords:** personality traits, psychosis, assessment, early intervention

## Abstract

*Background and Objectives*: The diagnosis of psychosis is a challenge for the scientific community, both in terms of its definition and treatment. Some recent studies have investigated the relationship between personality and psychosis onset to prevent or intervene early. *Materials and Methods*: Sixty young adults were recruited during their first access in 2019 near the Community Mental Health Service of Niguarda Hospital, Milan, Italy. The assessment included the Social and Occupational Functioning Assessment Scale (SOFAS), the Global Assessment of Functioning (GAF) (clinician scales), the 16-item Version of the Prodromal Questionnaire (PQ-16), the Personality Inventory for DSM-5 (PID-5) (self-report), and a clinical session. Statistical analysis was performed by SPSS. *Results*: The results show a negative correlation between the Detachment domain and the GAF scores. Correlational analysis also highlights that all PID-5 domains, except for Antagonism, have positive correlations with high scores in the PQ-16. The multivariate analysis of variance showed that patients diagnosed with versus without a psychotic disorder significantly differed on Detachment, Antagonism and Psychoticism PID-5 domains. *Conclusions*: The involvement of the personality construct in psychopathological development is displayed. In particular, higher levels of Detachment and Psychoticism can distinguish people who are more vulnerable to psychosis or who already have overt psychosis from those who do not have a psychotic predisposition. The study highlights the fundamental role of personality traits, emerging from PID-5, to distinguish young adults at risk of onset.

## 1. Introduction

Early intervention in mental health, initially born to respond quickly to the care needs of the first psychotic episode, has expanded by developing criteria, assessment tools and self-report screening including the Prodromal Questionnaire (PQ) [1] for the identification of subjects at high risk of psychosis.

Criteria had to be developed for “at risk state mental state” (ARMS). Different research groups have developed different early detection instruments and operationalization criteria. In 1994, the Personal Assessment and Crisis Evaluation (PACE) Clinic in Melbourne (Australia) started to develop the Ultra High-Risk (UHR) approach. This approach is based on identifying risk factors, trait or state factors, to be associated with an increased risk of psychosis disorders within a year. Classified as trait factors are having a schizotypal personality disorder or family history of psychotic illness; as state factors, experiencing mental distress and deteriorating functioning. The aim is to identify symptoms that are often seen before the onset of psychosis and focus on the age range (15–25 years) with the highest incidence of onset of psychotic disorders [2].

The criteria for UHR consist of three groups with specific conditions, one or more of which must be met, plus a significant decline or low functioning assessed using the Social and Occupational Functioning Scale (SOFAS) [3]. The three groups are: (1) Vulnerability group - having schizotypal personality disorder or a first-degree relative with a psychotic disorder, and having experienced a significant decrease in functioning during the previous year; (2) Attenuated psychotic symptoms group (APS) - have experienced sub-threshold, attenuated psychotic symptoms during the past year; (3) Brief limited intermittent psychotic symptoms group (BLIPS) - have experienced episodes of frank psychotic symptoms that have not lasted longer than a week and have been spontaneously resolved without treatment [4]. 

Where systematically implemented, early intervention is highly accessible and acceptable to young people and results in outcomes that are positive and cost-effective [2]. The early intervention paradigm has made substantial progress in developing early psychosis services and, more recently, specialized youth-specific services. Early intervention, particularly for subthreshold psychosis, has not occurred without controversy regarding the accuracy of detection of cases at risk of psychosis and the potential overtreatment of false positives. Only a small minority (36%) of cases considered with an at-risk mental state transits to psychosis after three years [5]. However, in early intervention services, a reduction in the rate of transition to psychosis has been suspected in recent years. That is to say, the UHR phenotype can have a number of different outcome trajectories, and early diagnosis increases the likelihood that we will identify those never intended to develop a psychotic disorder. Many individuals who at one stage meet UHR criteria diverge from the path to psychosis: some may experience the resolution of symptoms and difficulties, others may develop non-psychotic disorders [6]. In subjects with emergent nonpsychotic disorder, the annualized incidence of psychotic disorder is about one third (3.9–13.1%) compared to that of nonpsychotic mental disorders [7]. However, compared to the general population, incidence of psychotic disorders may be higher. It also provides a rationale for the change trajectories for those with vulnerabilities to psychotic diseases and a motivation for providing services to those who need help who could not access them previously [5]. Young people who access early intervention services typically demonstrate the need for treatment before reaching the threshold for a traditional main psychiatric diagnosis; when functional disorders and warning signs of mental illness are present, early intervention becomes crucial to prevent or reduce the severity of any full-threshold disorder.

Although it is not yet possible to definitively predict who will develop psychosis, it is possible to improve prediction in samples. The early intervention paradigm has historically been concerned with delaying or preventing a psychotic onset based essentially on the presence of positive subthreshold symptoms and a family history of psychotic disorders. The implicit paradigm is to treat any positive subthreshold symptoms as a path to psychosis. However, the concept of transition is oversimplified and presented uncritically as evidence to support at-risk mental states [8]. Clinical experience shows that subjects considered to be at high risk of developing overt psychosis present, in addition to attenuated psychotic symptoms, a wide range of psychopathological and other characteristics.

Epidemiological research has shown that psychotic experiences are a marker of the severity of multidimensional psychopathology. The subgroup of UHR subjects in transition to psychosis showed more social anhedonia and abstinence, more bizarre thinking, and a lower baseline GAF score than the subgroup that does not transition to psychosis [9]. 

Although a multitude of factors have been identified that contribute to one’s susceptibility for conversion to psychosis, such as the presence of unusual content of thought, low functioning and functional decline [10,11,12], personality features [13] and personality disorders [14,15], how basic differences in multidimensional psychopathology can have a differential impact on course and outcomes is still unclear. Further studies are needed. 

It is still doubtful to what extent personality may influence the development of psychosis. We aimed to explore significant personality traits in individuals at Ultra High-Risk (UHR) for psychosis.

Psychopathology and personality can relate to one another in three different ways. First, personality and psychopathology can influence the presentation or appearance of one another (pathoplastic relationship); second, they can share a common, underlying etiology, referred to as a spectrum relationship. Finally, they can have a causal role in the development or etiology of one another [16]. It has been argued that premorbid personality in psychosis may either have a pathoplastic effect, interacting with clinical symptoms at the onset of psychosis, or represent a vulnerability marker for such condition during neurodevelopmental processes in adolescence and young adulthood [17].

We conducted a retrospective and cross-sectional study with the aim to explore if and how the PID-5 traits from the DSM-5 Alternative Dimensional Model [18] can discriminate against patients in a group of help-seeking individuals with different classifications of mental states. We also analyzed the relationship between PID-5 personality traits and psychotic vulnerability using the Prodromal Questionnaire-16 used for psychosis risk screening and with the Global Assessment of Functioning (GAF). 

## 2. Materials and Methods

### 2.1. Participants 

The final sample consists of 60 young help seeking patients (27 males and 33 females) selected from new patients in 2019 in the youth mental health service for Early Intervention (EI) of Niguarda Hospital in Milan, Italy, known as Programma2000. Referrals to Programma2000 arrive from institutionally mediated pathways (e.g., primary care, district Mental Health Services, school counseling, emergency rooms) but can be also self-referrals from spontaneously help-seeking individuals. The metropolitan area served by the service includes approximately 350,000 inhabitants. Programma2000 is specialized both in evaluation procedures and in its care program. The treatment is based on a complete, tailor-made and flexible intervention package. The program includes individual psycho-educational and motivational sessions, cognitive-behavioral psychotherapy, psycho-education and individual family support, therapeutic group activities (e.g., anxiety management, assertive and problem-solving training, etc.), social group activities and support interventions on employment, school, medication compliance and recreational planning [19,20].

The participants, at the time of their first admission to the Center, were aged 18–24 with an average of 20.17 years (SD = 1.98). Participants were all patients initially assessed and treated by the Programma2000 from January 2019 to December 2019. In regard to the classification of mental state, most participants did not show any psychotic vulnerability (*n* = 32), 11 patients had a vulnerability due to genetic factors, 8 a vulnerability due to the presence of Attenuated Psychotic Symptoms and finally 10 had already had a fully-blown Psychotic Episode.

### 2.2. Procedures

Data were collected during the routine assessment of the patients participating in the Programma2000, the early intervention service operating under the Health Authority of the Niguarda Hospital of Milan [19]. The study complies with the guidelines of the 1995 Declaration of Helsinki and its revisions [21]. Participants provided informed consent. All clients consented to their clinical data being used for research in an anonymized way in group statistics.

Sixty cases were analyzed from an initial list of 100 new patients. There were some exclusion criteria: all patients presenting a Mental retardation diagnosis (ICD codes F70–79) [22], Autism (ICD code F84) [22], patients who dropped-out before completing the assessment, and those who had requested only a consultation for a diagnosis. Patients’ data were retrieved from the medical records. Patients were deemed to be UHR for psychosis when they met with the criteria of the Personal Assessment and Crisis Evaluation (PACE) Clinic in Melbourne for the identification of young people at incipient or ‘ultra-high risk’ of developing a psychotic disorder [23,24,25]. Amongst the UHR patients another category proposed by the PACE Clinic group was the one characterized by Brief Limited Intermittent Psychotic Symptoms (BLIPS) [25]. However, there were no participants who could be placed in this category; hence it was not considered in the analysis. The classification of at-risk mental state was evaluated in three stages. First, all patients were screened with the Prodromal Questionnaire (PQ-16). Secondly, all patients were examined independently at the time of admission by two expert clinicians. Finally, the two clinical evaluators discussed the case and the scores in the PQ16 with the Programma2000 team.

### 2.3. Research Tools

All patients completed the assessment of the Programma2000 for the prevention, individuation, and treatment of severe mental disorders in youth. The assessment includes the tools described below and the Social and Occupational Functioning Assessment Scale, or SOFAS [3]. We decided to focus our analysis on the PID-5, GAF, and PQ-16. 

#### 2.3.1. Personality Inventory for DSM-5 (PID-5)

The Personality Inventory for DSM-5 (or PID-5) [26] is a self-report questionnaire. It was developed to assess the Criteria B (Pathological Personality Traits) in section III of the DSM-5 with the aim of adopting a dimensional and inferential-contextual approach. The section introduced an innovative way to approach the diagnostic process considering the subjectivity of the patient and his biopsychosocial context [18]. 

It has 220 items with a Likert scale from 0 (Very False or Often False) to 3 (Very True or Often True). It assesses 25 personality facets, sub-dimensions that can be combined to form 5 main trait domains. The scoring was conducted following the indications of Krueger et al. [26]. The PID-5 main domains were determined by calculating the average of the corresponding facet scores. Facet scores were determined by calculating the average of all corresponding item scores to which the participants had answered [26]. 

#### 2.3.2. Prodromal Questionnaire (PQ-16)

The Prodromal Questionnaire (or PQ-16) [1] is a self-report questionnaire that was developed to identify psychotic vulnerability and used for psychosis risk screening. It evaluates the presence of negative and positive psychotic symptoms, considering the patient’s personal experience in the last month. The cut-off for psychotic vulnerability is a score of 6 (or more) [1]. The PQ-16 has 16 items that investigate different vulnerability aspects: nine items explore perceptual abnormalities and/or hallucinations, five items unusual thought content, delusional ideas and/or paranoia, and two items negative symptoms. For each item, the individual can indicate if they have experienced the symptom or not and then express the distress that experiencing that symptom entails, from 0 (No) to 3 (Severe). Scores of each item are added to arrive at the final score [1]. 

#### 2.3.3. Global Assessment of Functioning (GAF)

In Psychiatry, the severity of illness can be assessed by the Global Assessment of Functioning (or GAF) [27]. The GAF is globally recognized. It is structured as a comprehensive assessment of the global functioning of a patient, rating the functioning in the individual psychology, social and occupational areas, from positive mental health to severe psychopathology [28].

The GAF was designed to be a generic rating system, not linked to any specific diagnosis. It has a 100-point scale, divided into 10 intervals of 10 points each. Each interval has written instructions and examples to help the evaluation of the patient’s functioning. Higher scores reflect a healthier functioning of the patient, whilst lower scores indicate a patient with more severe illness [27].

### 2.4. Data Analysis

SPSS Statistics version 25 was used for data analysis. Firstly, the sample of participants was analyzed descriptively concerning sex, age, level of education and classification of At-Risk Mental State. To test the first hypothesis correlational analysis (with Pearson’s correlation coefficient) was conducted between the GAF [27] and PID-5 domains [26], together with correlations between domains and facets of the PID-5 and the scores of PQ-16 [1]. Lastly, to test the second hypothesis, a multi-variate analysis of variance (MANOVA) was conducted considering the classification of the mental state as a factor and the PID-5 domains as variables. To test the homogeneity of group variances the Levene test was conducted, whilst for the Post-Hoc analysis of the MANOVA the Tukey test was used. However, since in the MANOVA the Levene test was significant for the Psychoticism domain, the Welch test was also conducted for that domain, together with the Games-Howell test for the Post-Hoc analysis. 

## 3. Results

The sample comprised 60 patients. As shown in Table 1, they were aged 18–24 with a mean age = 20.17 (SD = 1.98) and were approximately 45% (=27) male and 55% (=33) female. According to the classification of mental state, 31 people had no psychotic vulnerability, 11 had psychotic vulnerability (with familiarity or disorder), 8 had an attenuated psychosis and 10 suffered from psychosis and had an antipsychotic treatment. 

### 3.1. Results from Correlation Analysis

We first conducted correlation analysis to evaluate clinically significant personality dimensions using traits in a group of help-seeking individuals with at-risk mental state and compared them to subjects at the first psychotic episode (FEP) and subjects who do not have psychotic predisposition. We also analyzed the relationship of clinically significant personality traits with functioning, assessed by the Global Assessment of Functioning (GAF), and possible transitions to psychosis in UHR individuals.

Between the GAF scale and the PID-5 domains, we used an alpha of 0.05 to determine statistical significance for these correlations. A relationship of mild strength and negative directions (r = −0.282, *p* = 0.029) emerged between the Detachment domain and the GAF scores, meaning that at higher scores in the Detachment domain, the GAF score decreases.

There were no other significant correlations between the PID-5 domains and the GAF scale. These results are shown in Table 2. 

In the correlation between PQ-16 and PID-5 domains, four of the five domains were most highly correlated. In this case, we used a conservative alpha of 0.001 to determine statistical significance. As shown in Table 3, all PID-5 domains, with the exception of Antagonism, were significantly correlated with a positive direction with the PQ-16 scores, in particular: Negative Affectivity (r = 0.548, *p* = 0.000), Detachment (r = 0.470, *p* = 0.000), Disinhibition (r = 0.501, *p* = 0.000) and finally, Psychoticism (r = 0.661, *p* = 0.000). 

Of the 25 PID-5 facets, 16 were significantly correlated with PQ-16 scores. For the Negative Affectivity domain, there was a positive correlation for alpha = 0.01 with the facets of Anxiousness (r = 0.350; *p* = 0.006), Emotional Lability (r = 0.528, *p* = 0.000), Hostility (also relevant for Antagonism) (r = 0.520, *p* = 0.000) and Perseveration (r = 0.361, *p* = 0.005); for alpha = 0.05 with the facets of Restricted Affectivity (r = 0.316, *p* = 0.014), and Separation Insecurity (r = 0.312, *p* = 0.0015). 

There was a positive correlation for alpha = 0.01 between the facets of Anhedonia (r = 0.385, *p* = 0.002), Depressivity (r = 0.388, *p* = 0.002) and Suspiciousness (r = 0.659, *p*), all sub-dimensions of the Detachment domain.

However, unexpected results were found in regard to the Antagonism domain; although it does not directly correlate with the PQ-16 scores, its facet of Deceitfulness is significantly correlated for alpha = 0.05 (r = 0.283, *p* = 0.028). The Disinhibition facets that correlate with the PQ-16 scores for alpha = 0.01 were Impulsivity (r = 0.399, *p* = 0.002) and Irresponsibility (r = 0.349, *p* = 0.006), while for alpha = 0.05 there was correlation with Distractibility (r = 0.317, *p* = 0.014). Finally, for the Psychoticism domain, all the facets correlated for alpha = 0.01, in particular: Eccentricity (r = 0.598, *p* = 0.000), Perceptual Dysregulation (r = 0.469, *p* = 0.000) and Beliefs and Unusual Experiences (r = 0.614, *p* = 0.000).

### 3.2. Results from MANOVA 

The multivariate analysis of variance including all PID-5 domain scores revealed a main effect of the classification of at-risk mental states emerging on the PID-5 domains of Detachment and Antagonism, Wilks’s Λ = 0.470 F (3.56) = 3.023 *p* = 0.000; η^2^ = 0.223. Patients classified as psychotic report higher values than other groups in these three PID-5 domains. Significant differences were found for alpha < 0.01 between groups for Detachment, F (3, 56) = 7.175, *p* = 0.000; η^2^ = 0.278, and Antagonism, F (3, 56) = 5.713, *p* = 0.002; η^2^ = 0.234.

Post-hoc comparisons (with Tukey’s test for alpha = 0.05) revealed that there are significant differences between the means among the groups for the Detachment and Antagonism domains. Table 4 shows the means and standard deviations for the PID-5 domains in each group.

Patients who do not have psychotic vulnerability show a reduced Detachment score (M = 1.04, SD = 0.50) compared to patients who show vulnerability, both for the presence of attenuated psychotic symptoms (M = 1.56, SD = 0.44) and for psychotic vulnerability (genetic factors) (M = 1.72, SD = 0.43). 

Patients with psychotic vulnerability (M = 1.72, SD = 0.43) report slightly higher scores than patients classified as psychotic (M = 1.16, SD = 0.41)

Comparisons between groups for the Antagonism domain showed that there is a difference between patients without psychotic vulnerability, who have lower levels (M = 0.51, SD = 0.37) than patients with attenuated psychotic symptoms (M = 0.91, SD = 0.23) and patients with Psychosis (M = 0.94, SD = 0.42). 

In regard to Psychoticism, the Levene’s F test revealed that the homogeneity of variance assumption was not met (*p* = 0.04). As such, the Welch’s F test was used. An alpha level of 0.05 was used for the analyses that revealed a statistically significant main effect of mental state classification on Psychoticism, Welch’s F (3,4.566) = 4.566 *p* = 0.014, indicating that not all patients had same average score on the Psychoticism domain. Post hoc comparison, using the Games-Howell post hoc procedure, was conducted to determine which pairs of the mental state classification differed significantly. The Games-Howell test did not reveal a significant effect, probably due to the low number that constitutes each group according to the mental state classification. 

## 4. Discussion

We initially examined the relationship between the Global Assessment of Functioning scale (GAF) and the trait model proposed in the Alternative Model for DSM-5 personality disorders (PID-5). In particular, Detachment was the only significant domain negatively correlated with GAF scores. The link between the Global Functioning scale and the Detachment domain may not be surprising in the help-seeking population in which about half of the sample (46.6%) has either a vulnerability to psychosis or an outspoken psychotic onset. In line with the literature, subjects with attenuated psychotic symptoms had significantly poorer outcomes [29,30]. 

Detachment is associated with avoidance of social and emotional experiences, withdrawal from interpersonal interactions and limited hedonic capacity. These correlations also suggest that high scores in the Detachment domain could be an index of a pre-morbid manifestation of negative symptoms. 

Social withdrawal, one of the facets of the Detachment domain, represents a very common behavior in the prodromal phase of psychosis [31], as well as depressive symptoms [32]. Even Suspiciousness, another facet of Detachment, is typical in some young people that, feeling socially inadequate, tend to incur “thought distortion”, attributing their ideas and opinions about themselves to others; this tendency can easily evolve in a straight persecutory framework [33]. 

The avoidance behavior or social withdrawal observed in UHR subjects has been hypothesized as reflecting an attempt to address positive symptoms through the control of external stimuli, even symptoms of social anxiety [34,35] or a negative dimension of symptomatology. One hypothesis would see social withdrawal, sometimes early, as triggering a possible cycle of maintaining disorder and disability. Initially it would act as a negative reinforcement, consisting in an attempt to minimize exposure to stimuli that would elicit positive or distress symptoms; then it would become a positive reinforcement contributing to low functioning, also through the atrophy of pre-existing social skills and a decrease in the quality of life [36,37]. Negative symptoms, defined as decreases in emotion, motivation, and/or expressive behavior [38], are a feature of schizophrenia and the attenuated psychosis syndrome [39]. Studies confirm that negative symptoms predict a number of poor clinical outcomes and limit social and vocational attainment [40,41,42]. Negative symptoms are highly associated with functional results in subjects that meet UHR criteria [43].

The Detachment domain, according to the Alternative Model for DSM 5, is present in the Avoidant and Schizotypal Personality Disorder. Our results on Detachment would seem to be in line with the literature that considers the inclusion of the Avoidant Personality disorder as a Schizophrenia spectrum disorder [44]. According to some studies, Detachment is mildly manifested in the premorbid period as well as subthreshold negative symptomatology [45]. When in the literature Schizoid Disorder, which by definition has Detachment domain characteristics (although the disorder is not considered by the Alternative Model for DSM 5), was also considered, it was found that schizoid rather than schizotypal personality traits predicted conversion to psychosis in the UHR patient, mainly from deficit in social interaction [46]. 

Negative symptoms typically appear years before the onset of attenuated positive symptoms and are one of the first risk indicators [47] as well as being the reason why individuals come into initial contact with the treatment system [31]. They are persistent, rather than episodic, unlike positive symptoms [48]. Accurately assessing negative symptoms at the beginning of the prodromal period, in which young people enter the treatment system for the first time, can allow efforts to be made to prevent the transition to psychosis. Negative symptoms are widespread in the prodromal phase as also confirmed by the North American Prodrome Longitudinal Study (NAPLS), where 82% of CHR cases were evaluated as having one or more moderately severity negative symptoms [39]. The careful evaluation of negative symptoms and personality traits could better clarify when the manifestations of detachment domain are primary in subjects considered to be at high risk of psychosis and therefore precursors of negative symptoms, or secondary manifestations’ effects of the emergence of negative and/or positive symptoms. More in-depth and longitudinal studies are needed to explore these hypotheses.

In the correlation between the Prodromal Questionnaire (PQ-16) and PID-5, four of the five domains were most highly correlated with PQ16 scores with the exception of Antagonism. It also has the lowest average scores among all domains (as shown in Table 3). Examining the correlations at the facet level, Anhedonia, Depressiveness and Suspiciousness, facets of Detachment, were highly correlated. Even this data is not surprising. The ARMS patients present a high prevalence of anxiety and depressive disorders in addition to their attenuated psychotic symptoms. These symptoms may reflect core emotional dysregulation processes and delusional mood in prodromal psychosis. Clinical experience shows that subjects considered to be at high risk of developing confirmed psychosis present, as well as attenuated psychotic symptoms, a wide range of other psychopathological characteristics. The very first observations show that the request for help is often triggered, rather than by attenuated psychotic symptoms, by the presence of depressive symptoms, anxiety and the emergence of a worrying decay in social functioning [32]. 

In our sample Detachment results are greater in patients considered vulnerable to psychosis and in patients with attenuated psychosis; this aspect could highlight the risk of the presence of other non-psychotic psychological or psychiatric disorders. Fusar Poli and collaborators [48] in a study of 509 UHR subjects, recruited into the English service better known as OASIS and the Australian PACE service, observed that depressive symptoms were associated with higher scores in Comprehensive Assessment of At-Risk Mental States (CAARMS) domains: negative symptoms, behavioral changes and general psychopathology. In particular, the highest scores were in the relative subscales Anhedonia and Abulia/Apathy in the subscale aimed at investigating disorganized behaviors, and in the subscale Suicidality and Self-Harm. Anhedonia and negative symptoms in general, are considered predictors of a high risk of transition to a psychotic disorder and poor social functioning in the long term.

It is necessary to clarify that the symptoms in depressive comorbidity differ from corresponding trait-like or facet Depressiveness of the domain detachment by their recent onset or worsening; depressiveness is a stable feature compared to symptoms; the latter could capture state-like signs of emerging disorder, that allow the beginning of an indicated prevention. Subjects with high levels of depressiveness are more likely to experience isolated episodes of depressive disorders. Both symptoms and traits are susceptible to change. Intervening on symptoms could also change the traits in the long term. 

The analysis of variance (MANOVA) underlined the validity of the PID-5 in its ability to discriminate between patients diagnosed with psychotic versus nonpsychotic disorders.

Detachment scores are in continuity with the study by Drvaric et al. [49], in which higher levels of this domain distinguished the patients most at-risk of psychosis from those less vulnerable. In our sample, in particular, it emerges that lower levels of Detachment can distinguish people in the sample not at risk for psychosis from those who present a vulnerability, both caused by genetic reasons and by attenuated psychotic symptoms. 

Antagonism, descriptively presenting the lowest mean levels and not correlating with PQ-16, differs significantly between groups with different mental state classifications. It is a domain for which the results were not straightforward. It is possible that the presence of many subjects in the sample diagnosed with Personality Disorders, also comorbid with psychotic disorders, might partly explain these results. Also for Psychoticism, a significant difference emerged between the groups from MANOVA. Nonetheless, homogeneity of variances between groups cannot be assumed, probably due to the low number that constitutes the group of psychotic patients according to the at-risk mental state classification. The results, although doubtful regarding Psychoticism, would be in line with the literature, which considers schizotypy as an indicator of being prone to psychosis and, therefore, a precursor to schizophrenia-spectrum disorders [50,51].

Results of this research show preliminary evidence of the utility of the PID-5 in assessing pathological personality. There are two significant limitations to the current study. The most substantial limitation concerns the use of a not very large sample. A second limitation concerns the use of self-report measures, such as PQ-16, where the score is tied to what the patient reports, which introduces shared method variance. Two other limitations concern the absence of subjects under the age of 18 and lack of information about Duration of Untreated Illness (DUI), duration of the disease, psychopharmacological treatment and resistant patients, together with the retrospective and cross-sectional nature of the study.

Even in light of these limitations, the current study is innovative in its attempt to underline the importance of using the DSM-5 personality trait model to identify psychopathology

## 5. Conclusions

The concept of at-risk mental state has led to a paradigm shift in clinical research and practice over the last 25 years. It has been shown that the onset of a confirmed psychotic disorder can at least be delayed, and a new generation of transdiagnostic research is leading the way. In particular, it concurs on whether UHR criteria have a value, not only for subsequent psychotic disorders, but also for persistent and concurrent non-psychotic syndromes. At the same time, it was demonstrated in a recent review [7] that a population at risk of non-psychotic disorders has about a 4% chance of developing psychosis over the next three years, lower than UHR but much higher than the general population. The current challenge is to develop a transdiagnostic Clinical Staging model that is consistent with clinical trajectories and takes into account the comorbidity present in mental disorders. This new approach goes beyond psychosis, to capture a wider range of subsoil symptoms and confirmed disorders; thus, it improves the possibility of predicting the emergence and progression of a number of mental disorders, as well as providing new ways of intervention. As we have seen before, psychotic disorders can emerge from other precursor states that are still undefined. There is retrospective evidence that some individuals may develop the First Episode of Psychosis without going through an identifiable Clinical High Risk (CHR) period [8]. One implication is that if most young people considered CHR do not develop a psychotic disease, and there are individuals who can develop psychosis without going through the CHR stage, mainly characterized by adhered positive symptoms, then the CHR stage, although the most likely, may not be the only route for psychotic disorders. Identifying maladaptive personality traits could help to better understand the ARMS group and how these can be factors that contribute to the risk of transition, but also better define the help-seeking group at low risk of transition, carriers of discomfort or symptoms that, regardless, need to be identified and promptly treated.

We believe the personality-related distinctions, observed in the current research, provide useful clinical information for the early treatment of those who may be at higher risk of conversion to psychosis. Considering the low transition rate in ARMS subjects, personality traits could be considered not only for their predictive value of converting to psychosis, but also as factors that contribute to the mental state at risk. The detection of clinically significant personality traits is paramount because it will improve our ability to accurately chart mental illness trajectories and determine when, where, and how to intervene more effectively to prevent serious and debilitating illness. From our results the Detachment domain seems to distinguish high-risk subjects from those at low risk of psychosis; this could be considered in the construction of treatment pathways and individualized treatments. Moreover, higher levels of Detachment were correlated with lower levels of functioning in our sample, which may suggest the importance of considering this information when planning treatment for early intervention. 

In future research, it would be important to examine participants ‘at-risk’ status at baseline and at a 2–3 years follow-up, comparing those who converted to psychosis and non-converters, while simultaneously examining positive and negative symptoms, social and role functioning, and neurocognitive domains in combination with maladaptive personality traits. 

## Figures and Tables

**Table 1:** Descriptive statistics of the sample.

Characteristic	Group	*n* (%)
Gender	Male	27 (45%)
	Female	33 (55%)
Age		60 (100%)
M (SD)	20.10 (2.07)	
Min-Max	16–24	
Education	No title	0 (0%)
	Primary school diploma	0 (0%)
	Middle school diploma	31 (51.7%)
	High school diploma	23 (38,3%)
	Graduate	6 (10%)
Work Status	No occupation	11 (18.34%)
	Full time occupation	2 (3.34%)
	Part-time occupation	0 (0%)
	Seasonal occupation	5 (8.33%)
	Occasional occupation	28 (46.66%)
	Student	14 (23.33%)
	Working student	0 (0%)
	Self-emplyed	0 (0%)
	Other	0 (0%)
Substance abuse	No	46 (76.67%)
	Yes	14 (23.33%)
Family history of a major mental disorder	No	31 (51.7%)
	Yes	29 (48.3%)
Classification of the mental state	No psychotic vulnerability	31 (53.3%)
		11 (18.3%)
	Psychotic vulnerability (with genetic factors)	
	Attenuated psychosis	8 (13.3%)
		10 (15%)
	Psychosis/antipsychotic treatment	

**Table 2:** The Pearson correlations between Global Assessment of Functioning (GAF) scale and PID-5 domains.

	GAF	
	Pearson Correlation	*p*
Negative Affect	−0.190	0.146
Detachment	−0.282 *	0.029
Antagonism	−0.144	0.272
Disinhibition	−0.118	0.370
Psychoticism	−0.87	0.506

* The correlation is significant at the 0.05 level.

**Table 3:** The Pearson correlations between PQ16 scale and PID-5 domains and facets.

	PQ-16	
	Pearson Correlation	*p*
**Negative Affect**	0.548 **	0.000
Anxiousness	0.350 **	0.006
Emotional Lability	0.528 **	0.000
Hostility	0.520 **	0.000
Perseveration	0.361 **	0.005
Restricted Affectivity	0.316 *	0.014
Separation Insecurity	0.312 *	0.015
Submisveness	0.136	0.300
**Detachment**	0.470 **	0.000
Anhedonia	0.385 **	0.002
Depressivity	0.388 **	0.002
Intimacy Avoidance	0.113	0.391
Suspiciousness	0.659 **	0.000
Withdrawal	0.204	0.119
**Antagonism**	0.214	0.100
Attention seeking	0.224	0.085
Callousness	0.215	0.099
Deceitfulness	0.283 *	0.028
Grandiosity	−0.014	0.918
Manipulativeness	0.055	0.677
**Disinhibition**	0.501 **	0.000
Distractibility	0.317 *	0.014
Impulsivity	0.399 **	0.002
Irresponsibility	0.349 **	0.006
Risk Taking	0.252	0.052
Perfectionism	0.197	0.131
**Psychoticism**	0.661 **	0.000
Unusual Beliefs and Experiences	0.641 **	0.000
Eccentricity	0.598 **	0.000
Perceptual Dysregulation	0.496 **	0.000

* The correlation is significant at the 0.05 level; ** The correlation is significant at the 0.01 level.

**Table 4:** Means and Standard Deviations of PID-5 Domains in Each Group.

	Classification	*n*	Mean	SD	*p*-Value	Post-Hoc
Negative Affect	No Vulnerability	31	1.30	0.41		
	Psychotic Vulnerability	11	1.59	0.41		
	Attenuated Psychosis	8	1.65	0.47		
	Psychosis/Antipsychotic treatment	10	1.40	0.46		
	Total	60	1.41	0.44		
Detachment	No Vulnerability	31	1.04	0.50	0.000 ^a^	(1) vs. (2) 0.001
	Psychotic Vulnerability	11	1.72	0.43		(2) vs. (4) 0.043
	Attenuated Psychosis	8	1.56	0.44		(1) vs. (3) 0.03
	Psychosis/Antipsychotic treatment	10	1.16	0.41		
	Total	60	1.25	0.53		
Antagonism	No Vulnerability	31	0.51	0.37	0.002 ^a^	(1) vs. (3) 0.025
	Psychotic Vulnerability	11	0.57	0.30		
	Attenuated Psychosis	8	0.91	0.23		
	Psychosis/Antipsychotic treatment	10	0.94	0.42		(1) vs. (4) 0.006
	Total	60	0.65	0.39		
Disinhibition	No Vulnerability	31	1.06	0.37		
	Psychotic Vulnerability	11	1.20	0.51		
	Attenuated Psychosis	8	1.39	0.32		
	Psychosis/Antipsychotic treatment	10	1.32	0.37		
	Total	60	1.17	0.40		
Psychoticism	No Vulnerability	31	0.70	0.41	0.014 ^b^	
	Psychotic Vulnerability	11	1.09	0.61		
	Attenuated Psychosis	8	1.10	0.34		
	Psychosis/Antipsychotic treatment	10	1.30	0.64		
	Total	60	0.93	0.53		

Classification of mental state = 1 = No vulnerability, 2 = Psychotic. vulnerability, 3 = Attenuated psychosis, 4 = Psychosis/Antipsychotic treatment; ^a^. *p*-value evaluated trough multi-variate analysis of variation (MANOVA); ^b^. Welch test.

## Data Availability

The data presented in this study are available on request from the corresponding author.
